# Chondrogenic potential of human articular chondrocytes and skeletal stem cells: A comparative study

**DOI:** 10.1177/0885328214548604

**Published:** 2014-08-20

**Authors:** Siwei Li, Bram G Sengers, Richard OC Oreffo, Rahul S Tare

**Affiliations:** 1 Centre for Human Development, Stem Cells and Regeneration, Faculty of Medicine, University of Southampton, Southampton, UK; 2 Faculty of Engineering and the Environment, Bioengineering Science, University of Southampton, Southampton, UK

**Keywords:** Articular chondrocytes, skeletal stem cells, pellets, scaffolds, hyaline cartilage, chondrogenesis, hypertrophy

## Abstract

Regenerative medicine strategies have increasingly focused on skeletal stem cells (SSCs), in response to concerns such as donor site morbidity, dedifferentiation and limited lifespan associated with the use of articular chondrocytes for cartilage repair. The suitability of SSCs for cartilage regeneration, however, remains to be fully determined. This study has examined the chondrogenic potential of human STRO-1-immunoselected SSCs (STRO-1^+^ SSCs), in comparison to human articular chondrocytes (HACs), by utilising two bioengineering strategies, namely “scaffold-free” three-dimensional (3-D) pellet culture and culture using commercially available, highly porous, 3-D scaffolds with interconnected pore networks. STRO-1^+^ SSCs were isolated by magnetic-activated cell sorting from bone marrow samples of haematologically normal osteoarthritic individuals following routine hip replacement procedures. Chondrocytes were isolated by sequential enzymatic digestion of deep zone articular cartilage pieces dissected from femoral heads of the same individuals. After expansion in monolayer cultures, the harvested cell populations were centrifuged to form high-density 3-D pellets and also seeded in the 3-D scaffold membranes, followed by culture in serum-free chondrogenic media under static conditions for 21 and 28 days, respectively. Chondrogenic differentiation was determined by gene expression, histological and immunohistochemical analyses. Robust cartilage formation and expression of hyaline cartilage-specific markers were observed in both day-21 pellets and day-28 explants generated using HACs. In comparison, STRO-1^+^ SSCs demonstrated significantly lower chondrogenic differentiation potential and a tendency for hypertrophic differentiation in day-21 pellets. Culture of STRO-1^+^ SSCs in the 3-D scaffolds improved the expression of hyaline cartilage-specific markers in day-28 explants, however, was unable to prevent hypertrophic differentiation of the SSC population. The advantages of application of SSCs in tissue engineering are widely recognised; the results of this study, however, highlight the need for further development of cell culture protocols that may otherwise limit the application of this stem cell population in cartilage bioengineering strategies.

## Introduction

Regeneration of articular cartilage, damaged as a result of trauma, injury or degeneration, remains a major challenge in Orthopaedics. Currently, there are no effective pharmacological agents that promote comprehensive healing of articular cartilage defects. The development of cell-based therapies to augment cartilage repair has therefore received considerable interest in recent years. Autologous chondrocyte implantation (ACI) is a widely used clinical intervention for the functional restoration of articular cartilage defects.^1^ For a typical two-stage ACI procedure, articular chondrocytes are harvested from the cartilage biopsy taken from the non weight-bearing region of the damaged joint, expanded in 2-D monolayer cultures and re-injected into the defect site under a periosteal flap.^1^ Although ACI has demonstrated promising clinical results,^2–4^ there are a number of limitations associated with the application of human articular chondrocytes (HACs) in this technique, namely donor site morbidity, limited source of cells, dedifferentiation of chondrocytes as a consequence of expansion in monolayer cultures and their limited lifespan *in vitro.*^5–7^ Hence, there is a pressing need for alternative effective cell sources to improve cartilage repair.

Skeletal stem cells (SSCs), synonymously referred to as mesenchymal stem cells (MSCs) derived from postnatal bone marrow stromal tissue, have attracted much attention due to their extended self-renewal potential and ability to differentiate into multiple stromal cell lineages.^8,9^ Bone marrow MSCs can be induced to differentiate into chondrocytes *in vitro* by utilisation of 3-D culture strategies in combination with chondroinductive factors (e.g. TGF-β3) and growth supplements.^10^ In comparison to autologous articular chondrocytes, application of autologous bone marrow-derived MSCs for articular cartilage repair is associated with one less surgical procedure, therefore reduced economic costs and minimal donor-site morbidity, and good to comparable clinical outcomes for cartilage repair.^11–13^ However, high variability in the chondrogenic differentiation potential of SSCs from different individuals, coupled with reports of generation of mechanically inferior fibrocartilaginous repair tissue by SSCs and tissue calcification, have limited the use of this adult stem cell population for cartilage regeneration.^14–16^ It is therefore debatable whether SSCs possess all the desirable characteristics to surpass, and hence replace articular chondrocytes in articular cartilage regeneration strategies.

Thus, SSCs and HACs obtained from the same osteoarthritic individuals were compared in the present study for their potential to generate hyaline cartilage-like tissue, utilising two tissue engineering strategies, namely “scaffold-free” pellet culture and culture in highly porous 3-D Alvetex® scaffolds.

## Materials and methods

Chemicals and reagents were purchased from Invitrogen (Paisley, UK) and Sigma-Aldrich (Gillingham, UK) unless specified. Human bone marrow and femoral head samples were obtained from eight haematologically normal osteoarthritic individuals (three males and five females, mean age: 80 ± 14 years) following routine total hip replacement surgery at Southampton General Hospital. Only tissue that would have been discarded was used in this study with approval of the Southampton and South West Hampshire Research Ethics Committee (Ref No. 194/99/1 & 210/01).

### Isolation of STRO-1-immunoselected SSCs

Following extraction of cells from bone marrow samples in α-MEM, the cell suspension was gently layered over Lymphoprep (Axis-Shield Diagnostic, Dundee, UK) and centrifuged to remove red blood cells by sedimentation. Bone marrow mononuclear cells (BMMNCs) collected from the ‘buffy coat’ at the interphase were incubated with the mouse monoclonal STRO-1 antibody (undiluted supernatant harvested from the STRO-1 hybridoma in-house), and the SSC-enriched STRO-1^+^ cell population was isolated by MACS as described previously.^17^ STRO-1^+^ cells were cultured to confluence in monolayer cultures in basal medium (α-MEM supplemented with 10% (v/v) FCS, 100 unit/ml penicillin and 100 µg/ml streptomycin); cultures were maintained in humidified atmosphere at 37℃, 5% CO_2_ and 21% O_2_. Passage 2 cells were utilised for the experiments.

### Isolation of HACs

HACs were isolated by sequential enzymatic digestion of deep-zone articular cartilage pieces, dissected from the non load-bearing region of the femoral heads.^18^ In brief, cartilage pieces were sequentially digested with 500 µg/ml trypsin-EDTA for 30 min, 1 mg/ml hyaluronidase for 15 min and 10 mg/ml collagenase B (ROCHE Diagnostics, Burgess Hill, UK) on a rotating mixer overnight at 37℃. Isolated chondrocytes were cultured to confluence in monolayer cultures in α-MEM supplemented with 10% (v/v) FCS, 100 unit/ml penicillin, 100 µg/ml streptomycin and 100 μM ascorbate 2-phosphate. Cultures were maintained in humidified atmosphere at 37℃, 5% CO_2_ and 21% O_2_. Passage 1 cells were utilised for the experiments.

### Pellet culture

Pellet cultures were performed in accordance with the protocol published previously.^19^ HACs and STRO-1^+^ SSCs were harvested at confluence from monolayer cultures and suspended in serum-free chondrogenic media at final concentrations of 0.6 × 10^5^, 1 × 10^5^, 2 × 10^5^, 3 × 10^5^ and 5 × 10^5^ cells/ml. 1 ml of cell suspension was added to each sterile 25 ml polycarbonate universal tube and centrifuged at 400 × g for 5 minutes at 4℃. The resulting cell pellet was cultured in humidified atmosphere at 37℃, 5% CO_2_ and 21% O_2_ for 21 days. The serum-free chondrogenic medium was made up of α-MEM supplemented with 10 ng/ml rhTGF-β3 (PeproTech, London, UK), 100 μM ascorbate-2-phosphate, 10 nM dexamethasone and 1X ITS liquid supplement (10 µg/ml insulin, 5.5 µg/ml transferrin and 5 ng/ml selenite premix), and media changes were carried out every two days over the 21-day culture period. For each cell type, 2–5 pellets were generated using each of the above mentioned cell numbers. At the end of the culture period, three pellets (generated using 3 × 10^5^ HACs and STRO-1^+^ SSCs) were harvested for analysis of chondrogenic gene expression, while two pellets from each group were fixed in 4% paraformaldehyde (PFA) and used for histological examination.

### Cell culture in Alvetex® 3-D scaffold inserts

Commercially available Alvetex® 3-D scaffold membranes mounted in inserts suitable for 12-well plates (Amsbio, Abingdon, UK; Cat. # AMS.AVP005-34) were used in this study. The Alvetex® scaffold inserts were prepared according to manufacturer’s instructions. In brief, the scaffold inserts were initially immersed in 70% ethanol for less than 1 min and then washed thoroughly with PBS, prior to incubation in plain α-MEM for 15 minutes in a CO_2_ incubator. The scaffold inserts were moved into 12-well plates, and 1 × 10^6^ cells suspended in 3.5 ml chondrogenic medium were gently added onto each scaffold membrane and the plates were returned to humidified atmosphere at 37℃, 5% CO_2_ and 21% O_2_. 3–5 scaffold inserts were seeded with each cell type per patient. The inserts were not disturbed for the initial three days to allow cell attachment and chondrogenic media were changed thereafter every three days during the 28-day culture period. At the end of the culture period, three explants were harvested for analysis of chondrogenic gene expression, while two explants from each group were fixed in 4% paraformaldehyde (PFA) and used for histological examination.

### Analysis of gene expression

Three day-21 pellets/day-28 explants from each group were pooled and lysed using 20-gauge needles for extraction of total RNA using Qiagen RNeasy kit (Qiagen, Manchester, UK) following manufacturer’s instructions. The RNA samples were treated with DNase-1 reagent and reverse-transcribed using the SuperScript® VILO™ kit (Invitrogen, Paisley, UK). qPCR assays were carried out using the 7500 Real-Time PCR system (Applied Biosystems, Paisley, UK) for analysing the expression of chondrogenic genes, namely *Sox-9* (*NM_000346* – F: 5′ CCCTTCAACCTCCCACACTA 3′; R: 5′ TGGTGGTCG GTGTAGTCGTA 3′), *Col2a1* (*NM_001844, NM_033150* – F: 5′ CCTGGTCCCCCTGGTCTTGG 3′; R: 5′ CATCAAATCCTCCAGCCATC 3′), *Aggrecan* (*NM_001135, NM_013227* – F: 5′GACGGCTTCCACCAG TGT 3′; R: 5′ GTCTCCATAGCAGCCTTCC 3′) and *Col10a1* (*NM_000493* – F: 5′ CCCACTACCCAACACCAAGA 3′; R: 5′GTGGACCAGGAGTACCTTGC 3′). The expression of genes of interest was normalised to the housekeeping gene/endogenous control, *β-actin* (*NM_ 001101* – F: 5′ GGCATCCTCACCCTGAAGTA 3′; R: 5′ AGGTGTGGTGCCAGATTTTC 3′). Since the SYBR Green method was used, primers were validated by dissociation curve/melt curve analysis to rule out the formation of primer dimers. The relative transcript levels of genes of interest were analysed using the comparative C_T_ method (ΔΔC_T_ method). For a valid ΔΔC_T_ calculation, the efficiencies of target (gene of interest) and reference (endogenous control) amplification were confirmed to be equal. For each gene, the group with the highest expression was assigned a value of 1 and expression level in the other group was determined as relative fold decrease. Fold relative transcript levels were expressed as mean ± SD for plotting as bar graphs. Statistical analysis was performed at the level of ΔC_T_ in order to exclude potential bias due to averaging of data transformed through the 2^− ΔΔC^_T_ equation.

### Histological analysis

PFA-fixed samples were processed through graded ethanol (50–100%) and histoclear (100%) prior to embedding in paraffin wax. Sequential sections were cut at 7 µm on the microtome and mounted on glass slides for histological and immunohistochemical staining. Images were captured with Olympus dotSlide virtual slide system (Olympus Microscopy, Southend-on-Sea, UK).

### Alcian blue and Sirius red (A/S) staining

Sections were stained with Alcian blue 8GX (5 mg/ml in 1% (v/v) glacial acetic acid) and Sirius red F3B (10 mg/ml in saturated picric acid) following nuclear staining with Weigert’s haematoxylin.^20^ Alcian blue stained the proteoglycan-rich cartilage matrix, while Sirius red stained the collagen-rich matrix.

### Immunohistochemistry

After quenching endogenous peroxidase activity with 3% (v/v) H_2_O_2_ and blocking with 10 mg/ml BSA in PBS, sections were incubated with relevant primary antiserum at 4℃ overnight. This was followed by hour-long incubation each with the appropriate biotinylated secondary antibody and ExtrAvidin®-Peroxidase. Visualisation of the immune complex involved the avidin-biotin method linked to peroxidase and AEC (3-amino-9-ethylcarbazole), resulting in a reddish brown reaction product. Negative controls (omission of the primary antisera) were included in all immunohistochemistry procedures. No staining was observed in all negative control sections. All sections were counter-stained with Alcian blue 8GX. The anti-SOX-9 antibody (rabbit polyclonal, IgG, Millipore, Watford, UK) was used at a dilution of 1:150 in 10 mg/ml BSA in PBS, following the antigen retrieval procedure, which involved heating sections in 0.01 M citrate buffer (pH 6.0) in a microwave for 5 min before the application of the standard immunohistochemistry procedure. For immunostaining using anti-collagen Type I, II and X antibodies, sections were treated with Hyaluronidase (0.8 mg/ml) at 37℃ for 20 min in order to unmask the collagen fibres and render them accessible for immunostaining. The LF68 anti-collagen Type I antibody (rabbit IgG, gift from Dr Larry Fisher), anti-collagen Type II antibody (rabbit IgG, Calbiochem, Watford, UK) and anti-collagen Type X antibody (rabbit IgG, Calbiochem, Watford, UK) were used at dilutions of 1:1000, 1:500 and 1:100, respectively.

### Statistical analysis

Results were presented as mean ± S.D. Statistical analysis was performed using Mann–Whitney U test. Results were deemed significant if the probability of occurrence by random chance alone was less than 5% (i.e. *p* < 0.05).

## Results

### Chondrogenic differentiation of HACs and STRO-1^+^ SSCs in scaffold-free pellet culture

**Figure 1.**
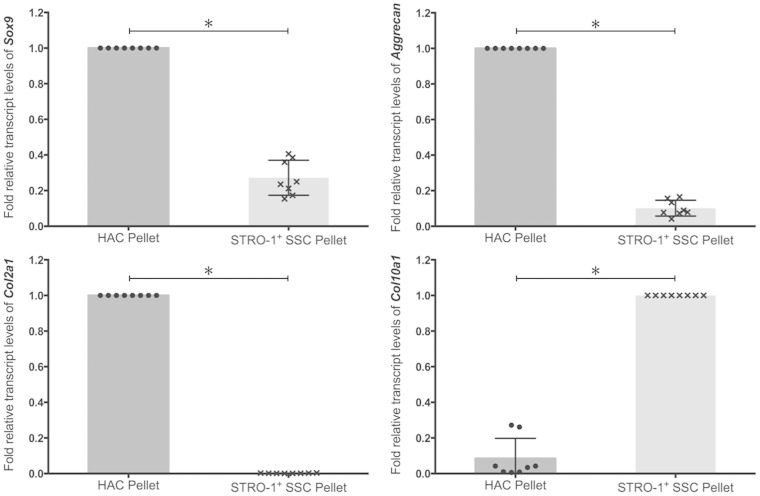
Analysis of expression of *Sox-9*, *Aggrecan*, *Col2a1* and *Col10a1* transcripts by real-time qPCR in day-21 pellets generated using 3×10^5^ HACs and STRO-1^+^ SSCs. Expression levels of the chondrogenic genes were normalised to the expression of the housekeeping gene, *β-Actin*. For each gene, the group with the highest expression was assigned a value of 1 and the expression level in the other group was determined as relative fold decrease. Results are expressed as mean ± SD; *n* = pellets from eight osteoarthritic individuals; *p < 0.05 (values of ΔC_T_ were used for statistical analysis).

Day-21 pellets of HACs and STRO-1^+^ SSCs were harvested for analysis of chondrogenic gene expression, and histological and immunohistochemical examination. Expression of chondrogenic genes in day-21 pellets generated using 3 × 10^5^ HACs and STRO-1^+^ SSCs was measured by qPCR (Figure 1). Although expression of *Sox-9*, a key marker of chondrogenesis, was detected in day-21 pellets of HACs and STRO-1^+^ SSCs, levels of the *Sox-9* transcript were significantly higher in HAC pellets compared to pellet cultures of STRO-1^+^ SSCs. Expression levels of *Col2a1*, the gene encoding collagen Type II – the hyaline cartilage-specific collagen, and *Aggrecan*, the gene encoding the major proteoglycan in hyaline cartilage, were significantly lower in pellet cultures of STRO-1^+^ SSCs compared to HACs. In contrast, expression of *Col10a1*, the gene encoding collagen Type X – a marker of terminally differentiated or hypertrophic chondrocytes, was significantly increased in pellet cultures of STRO-1^+^ SSCs compared to day-21 pellets of HACs.

**Figure 2.**
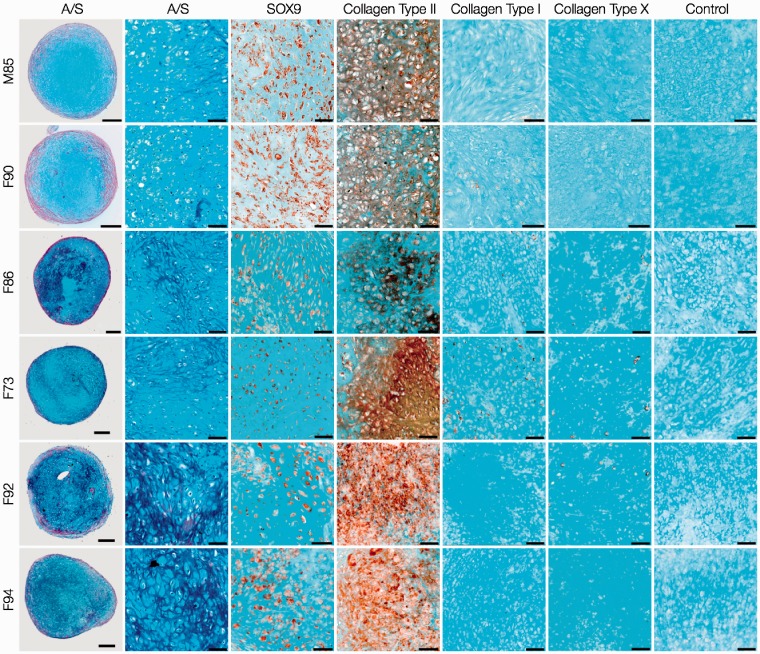
Histological and immunohistochemical examination of day-21 HAC pellet cultures. HACs from six osteoarthritic individuals (M85, F90, F86, F73, F92, F94) were cultured as 3-D pellets (3 × 10^5^ cells per pellet) over a period of 21 days in chondrogenic media. Sections stained with Alcian blue and Sirius red (A/S) demonstrated formation of hyaline cartilage-like tissue in all pellets. Chondrogenic differentiation was confirmed by robust expression of SOX-9 in chondrocytes and collagen Type II in chondrocytes and the surrounding extracellular matrix, coupled with negligible expression of collagen Type I and X. Scale bars for low and high magnification images represent 200 µm and 50 µm, respectively.

**Figure 3.**
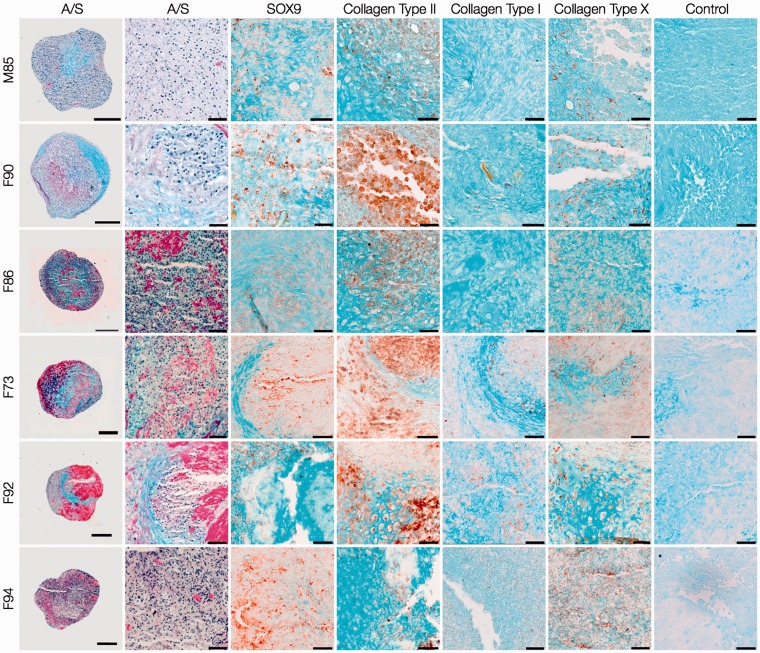
Histological and immunohistochemical analysis of day-21 STRO-1^+^SSC pellet cultures. STRO-1-immunoselected SSCs from the six osteoarthritic individuals (M85, F90, F86, F73, F92, F94) were cultured as 3-D pellets (3 × 10^5^ cells per pellet) over a period of 21 days in chondrogenic media. Sections stained with Alcian blue and Sirius red (A/S) demonstrated lack of hyaline cartilage-like tissue in all pellets. Staining for SOX-9 and collagen Type II in STRO-1^+^ SSC pellets was not extensive. Negligible staining for collagen Type I was accompanied by expression of collagen Type X in a large number of cells of the STRO-1^+^ SSC pellets. Scale bars for low and high magnification images represent 200 µm and 50 µm, respectively.

For analysis of cartilage formation and chondrogenic differentiation, sections of day-21 pellets generated using 3 × 10^5^ HACs (Figure 2) and STRO-1^+^ SSCs (Figure 3) were stained with Alcian blue and Sirius red (A/S), and immunostained with antibodies against phenotypic chondrogenic proteins. HAC pellets exhibited hyaline cartilage-like tissue, composed of chondrocytes in distinct lacunae embedded in dense proteoglycan matrix stained with Alcian blue. A layer of Sirius red-stained fibrous collagen was observed prominently along the periphery of the pellets. SOX-9 was immunolocalised to majority of the chondrocytes of the pellets, and prominent presence of collagen Type II was observed in the chondrocytes as well as the surrounding extracellular matrix. Furthermore, the pellets demonstrated negligible staining for collagen Type I, a constituent of fibrocartilage, and collagen Type X, a phenotypic marker of hypertrophic cartilage.

In comparison to HAC pellets, day-21 pellets of STRO-1^+^ SSCs demonstrated lack of hyaline cartilage-like tissue. Distinct regions of Sirius red-stained matrix adjacent to small areas of Alcian blue-stained cartilaginous tissue were observed in day-21 pellets of some STRO-1^+^ SSCs (F86, F73, F92). Staining for SOX-9 and collagen Type II in STRO-1^+^ SSC pellets was not as extensive as in day-21 pellets of HACs and complemented *Sox-9* and *Col2a1* gene expression. Negligible staining for collagen Type I was accompanied by expression of collagen Type X in a significant proportion of cells of the STRO-1^+^ SSC pellets that mimicked *Col10a1* gene expression. STRO-1^+^ SSCs therefore exhibited hypertrophic differentiation and were not able to generate robust hyaline cartilage-like tissue in day-21 pellets.

**Figure 4.**
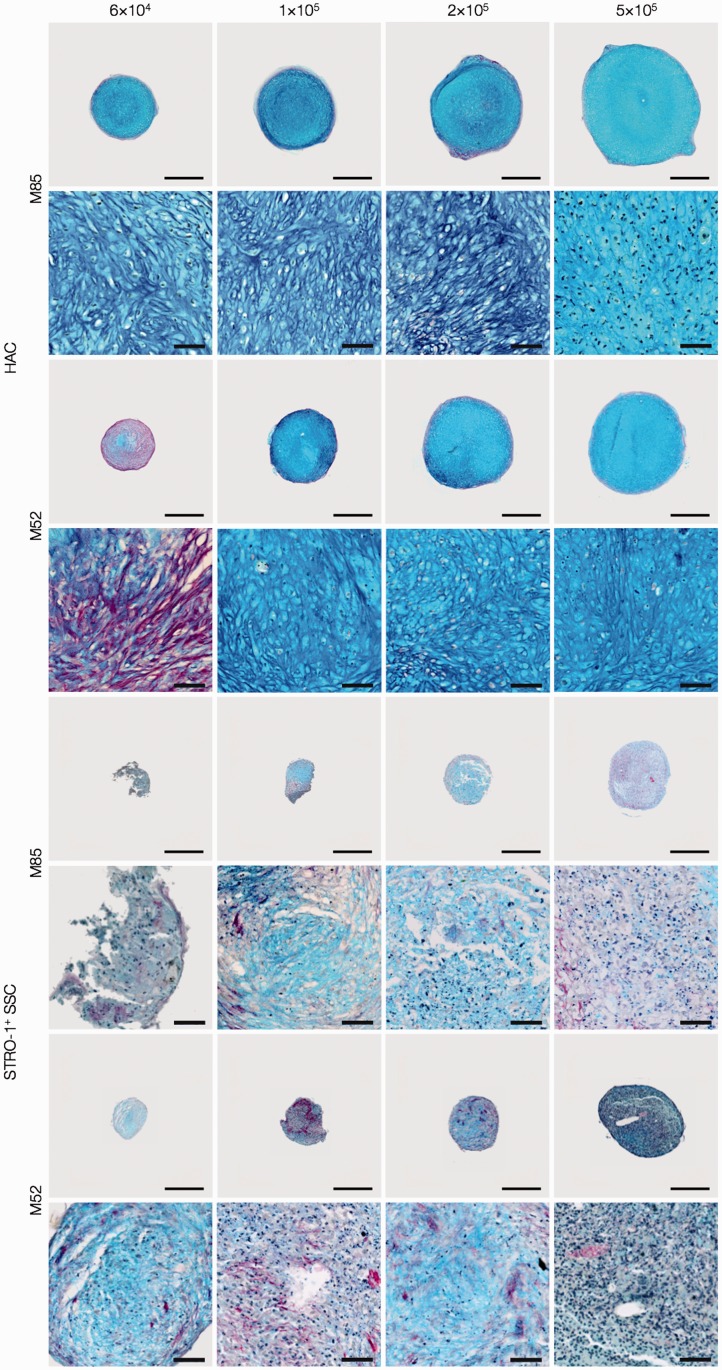
Examination of chondrogenic differentiation in day-21 pellets generated using 0.6 × 10^5^, 1 × 10^5^, 2 × 10^5^ and 5 × 10^5^ HACs and STRO-1^+^ SSCs from two osteoarthritic individuals (M85, M52). Histological sections of the day-21 pellets were stained with Alcian blue and Sirius red (A/S). Formation of hyaline cartilage-like tissue was observed consistently in day-21 pellets generated using 0.6 × 10^5^–5 × 10^5^ HACs, while day-21 pellets generated using 0.6 × 10^5^–5 × 10^5^ STRO-1^+^ SSCs demonstrated lack of hyaline cartilage formation. Scale bars for low and high magnification images represent 200 µm and 50 µm, respectively.

To determine whether suboptimal chondrogenic differentiation and cartilage formation were restricted to pellets generated using 3 × 10^5^ STRO-1^+^ SSCs, additional pellets were generated using 0.6 × 10^5^, 1 × 10^5^, 2 × 10^5^ and 5 × 10^5^ STRO-1^+^ SSCs from two osteoarthritic individuals (M85, M52). For comparison, similar cell numbers were used to generate pellets of HACs from the same individuals. Histological sections of the day-21 pellets were stained with A/S and examined for cartilage formation (Figure 4). Formation of robust hyaline cartilage-like tissue was observed consistently in day-21 pellets generated using 0.6 × 10^5^–5 × 10^5^ HACs, while day-21 pellets generated using 0.6 × 10^5^–5 × 10^5^ STRO-1^+^SSCs showed lack of hyaline cartilage formation.

### Generation of cartilaginous explants by HACs and STRO-1^+^ SSCs cultured in Alvetex® 3-D scaffolds

**Figure 5.**
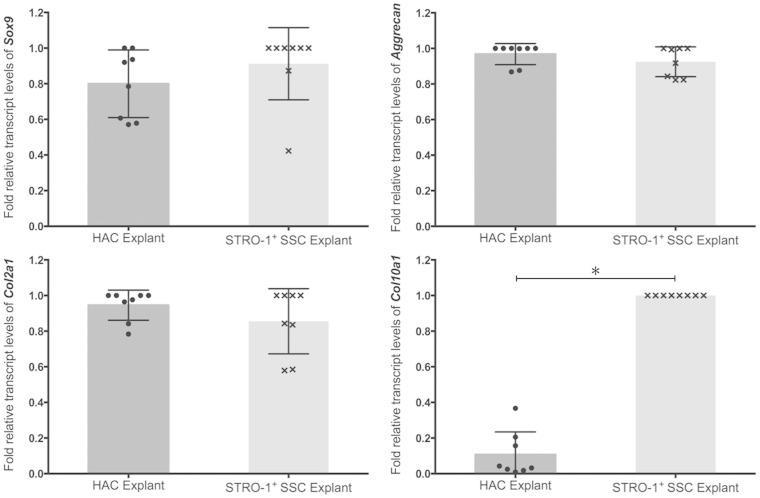
Analysis of expression of *Sox-9*, *Aggrecan*, *Col2a1* and *Col10a1* transcripts by real-time qPCR in day-28 explants of HACs and STRO-1^+^ SSCs cultured in Alvetex® 3-D scaffolds. Expression levels of the chondrogenic genes were normalised to the expression of the housekeeping gene, *β-Actin*. For each gene, the group with the highest expression was assigned a value of 1 and the expression level in the other group was determined as relative fold decrease. Results are expressed as mean ± SD; *n* = explants from eight osteoarthritic individuals; *p < 0.05 (values of ΔC_T_ were used for statistical analysis).

Following culture of HACs and STRO-1^+^ SSCs in Alvetex® 3-D scaffolds, expression levels of the key chondrogenic genes, namely *Sox-9*, *Aggrecan, Col2a1* and *Col10a1*, in day-28 explants were analysed by qPCR (Figure 5). Robust expression of *Sox-9*, *Aggrecan* and *Col2a1* was observed in day-28 explants of HACs. Interestingly, expression levels of *Sox-9*, *Aggrecan* and *Col2a1* in day-28 explants of STRO-1^+^ SSCs were comparable to day-28 HAC explants. Expression of the hypertrophic gene *Col10a1* in day-28 explants of STRO-1^+^ SSCs, however, remained significantly higher than day-28 HAC explants.

**Figure 6.**
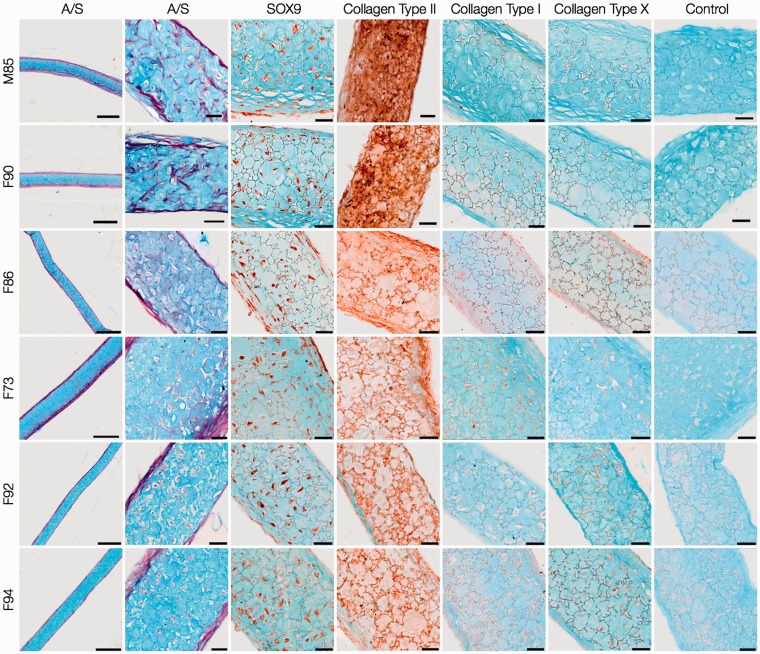
Histological and immunohistochemical analysis of day-28 HAC explants. HACs from six osteoarthritic individuals (M85, F90, F86, F73, F92, F94) were cultured in Alvetex® 3-D scaffolds for 28 days in chondrogenic media. Sections of the cartilaginous explants demonstrated presence of chondrocytes in lacunae embedded in Alcian blue-stained proteoglycan matrix surrounded by a thin layer of Sirius red-stained fibrous collagen along the periphery. Chondrogenic differentiation was confirmed by robust staining for SOX-9 and collagen Type II, and negligible staining for collagens Type I and Type X. Scale bars for low and high magnification images represent 200 µm and 50 µm, respectively.

**Figure 7.**
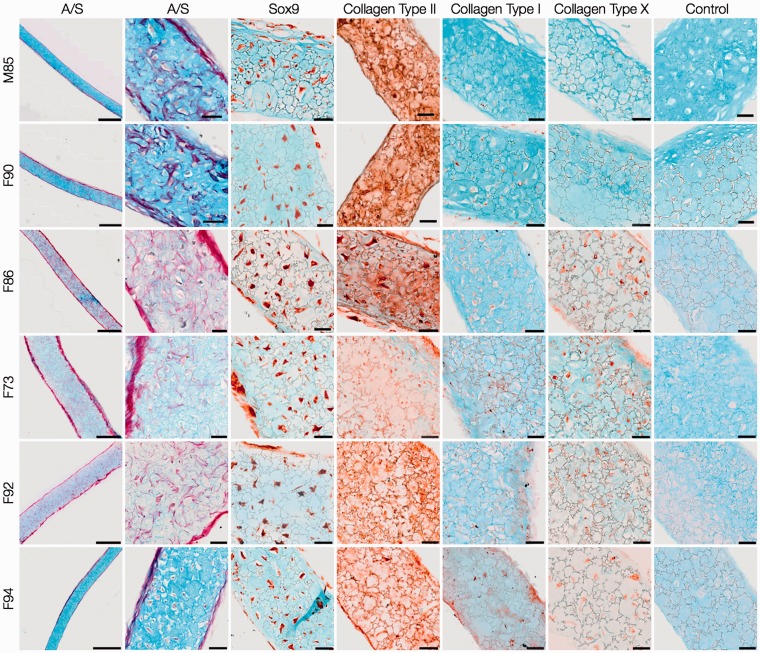
Histological and immunohistochemical examination of day-28 STRO-1^+^ SSC explants. STRO-1-immunoselected SSCs from six osteoarthritic individuals (M85, F90, F86, F73, F92, F94) were cultured in Alvetex® 3-D scaffolds for 28 days in chondrogenic media. The overall histology of the cartilaginous explants stained with Alcian blue and Sirius red (A/S) was comparable to that of HAC explants. Chondrogenic differentiation of STRO-1^+^ SSCs was significantly improved as demonstrated by the robust expression of SOX-9 and collagen Type II, and negligible staining for collagen Type I. Staining for collagen Type X was observed in some cells of the day-28 explants . Scale bars for low and high magnification images represent 200 µm and 50 µm, respectively.

Day-28 explants of HACs (Figure 6) and STRO-1^+^ SSCs (Figure 7) were characterised by the presence of chondrocytes in lacunae embedded in Alcian blue-stained proteoglycan matrix surrounded by a thin layer of Sirius red-stained fibrous collagen along the periphery. The expression of SOX9 confirmed robust chondrogenic differentiation in explants of both cell types. The collagenous matrix of day-28 explants of HACs consisted of collagen Type II, while negligible collagen Types I and X were observed. In addition to robust staining for collagen Type II and negligible expression of collagen Type I, staining for collagen Type X was observed in some cells of day-28 SSC explants.

## Discussion

The present study has investigated the chondrogenic potential of two distinct, but clinically relevant, cell populations, namely HACs and SSCs, using “scaffold-free” pellet culture and culture in Alvetex® 3-D scaffolds. To the best of our knowledge, this is the first study that has comprehensively analysed the chondrogenic differentiation of HACs and SSCs in Alvetex® scaffolds following long-term *in vitro* culture. Deep zone articular chondrocytes were utilised in this work, since previous studies have reported that, in osteoarthritis, degenerative changes are initiated in the superficial zone of articular cartilage and expression of matrix metalloproteinases, including MMP-2, MMP-3, MMP-11 and ADAMTS5, is significantly upregulated in superficial zone chondrocytes.^21–23^ The monoclonal antibody, STRO-1, capable of recognising the trypsin-resistant, cell-surface antigen expressed by a subset of bone marrow mononuclear cells enriched in stem/progenitor cells, was used in the present study to immunoselect the STRO-1^+^ SSC population from bone marrow samples.^24^ The STRO-1 antibody is one of the most widely used antibodies for selecting stem cell-enriched populations from bone marrow, adipose tissue, synovial tissue and dental pulp. The STRO-1^+^ cell population of human bone marrow is enriched in SSCs, which have the ability to transfer a functional hematopoietic microenvironment *in vitro* and are able to differentiate into multiple stromal cell types, including smooth muscle cells, adipocytes, osteoblasts and chondrocytes.^25^ Thus, by obtaining the two cell populations from each of the eight osteoarthritic individuals, the study was able to make a reliable comparison between the chondrogenic potential of the two distinct cell types.

The 3-D pellet culture model has been widely employed to stimulate chondrogenesis and generate scaffold-free cartilaginous explants that are characterised by morphological, gene and protein expression profiles matching *in vivo* hyaline cartilage.^10,19,26^ In the present study, while pellet culture served as an excellent *in vitro* system to facilitate the generation of hyaline cartilage-like tissue from monolayer-expanded, dedifferentiated HACs, STRO-1^+^ SSCs from the same eight osteoarthritic individuals exhibited hypertrophic differentiation and lacked the ability to generate hyaline cartilage in day-21 pellets. In a separate study, which examined the chondrogenic potential of articular chondrocytes and the heterogeneous bone marrow stromal cell (BMSC) population obtained from 30 osteoarthritic subjects, chondrocytes were shown to exhibit robust chondrogenic potential in micromass cultures compared to bone marrow stromal cells (BMSCs), which, unlike articular chondrocytes, expressed significantly higher levels of *Col1a1* and *Col10a1* in the micromass cultures.^27^ Likewise, lack of hypertrophy and terminal differentiation were observed in pellet cultures of monolayer-expanded HACs, in contrast to pellets of BMSCs, which demonstrated hypertrophy, extensive calcification and vascular invasion upon subcutaneous implantation in immunocompromised mice.^28^ Moreover, aggregates of the relatively homogeneous STRO-1-immunoselected SSC population cultured for 3 weeks in the presence of 10 ng/ml TGF-β1/3 were characterised by the robust expression of *Col10a1*/Type X collagen.^25,29^

Thus, results of the work presented here and in the aforementioned studies have demonstrated that STRO-1^+^ SSCs and BMSCs lack the ability to generate robust hyaline cartilage-like tissue in high density, scaffold-free, 3-D culture techniques, such as pellet, aggregate or micromass culture. This is because STRO-1^+^ cells have a lower chondrogenic differentiation potential compared to articular chondrocytes in the pellet culture system and a predisposition for hypertrophic differentiation even in the presence of TGF-β1/3. Although the STRO-1^+^ cell population is enriched in SSCs, which are able to differentiate into multiple stromal cell lineages, including the chondrogenic lineage, application of the STRO-1 antibody alone does not yield a homogenous population comprising solely of multipotent SSCs. This is because the STRO-1 antibody also selects other cell types from the bone marrow, including the osteoprogenitor subpopulation.^30^ It is therefore possible that the contaminating cell types within the heterogeneous STRO-1^+^ cell population, especially the osteoprogenitor cells, which are committed to differentiate into the osteogenic lineage, affect the chondrogenic differentiation of the SSC subpopulation of the STRO-1^+^ cell population. Moreover, it has been suggested that a hypertrophic chondrocyte phenotype typical of the transient cartilage observed in the process of endochondral ossification is the default lineage intrinsic to bone marrow-derived SSCs.^31^

Although articular chondrocytes undergo dedifferentiation during monolayer culture, this is a transient phenotype, as the cells are believed to retain their “developmental memory”, most likely mediated by epigenetic mechanisms such as DNA methylation, which assists in preventing hypertrophic differentiation, calcification and vascular invasion.^28^ Articular chondrocytes therefore retain their chondrogenic differentiation potential and produce hyaline cartilage-like tissue in the 3-D environment of the pellet culture system.

In comparison to pellet culture, the present study demonstrated a significant improvement in the expression of hyaline cartilage-specific markers by STRO-1^+^SSCs following culture in the Alvetex® 3-D scaffold membranes. *In vivo*, articular chondrocytes receive oxygen and nutrients mainly via diffusion from the capillaries of the synovium into the synovial fluid and then into the cartilage matrix.^32^ *In vitro*, mass transport limitations and oxygen diffusion gradients in the macroscopic pellets have been shown to be significant obstacles to robust chondrogenesis of bone marrow-derived MSCs.^33^ In contrast, highly porous scaffolds with interconnected pore networks promote cartilage formation due to improved infiltration of cells, enhanced mass transportation of oxygen and nutrients, and efficient clearance of metabolic wastes.^34,35^ Thus, porosity, pore size and interconnectivity are important architectural properties of a scaffold that influence production of cartilage matrix.^36^

The highly porous (around 90% porosity) Alvetex® 3-D scaffolds are made from inert polystyrene (a substrate widely used in cell culture) and are characterised by the presence of interconnected pore networks. It is likely that the relatively thin membrane-like structure (200 µm thickness), coupled with the highly interconnected and accessible pores of the Alvetex® scaffold contributed to the optimal diffusion of gases, nutrients and waste metabolites between the cells and culture medium during the prolonged culture period, and facilitated chondrogenic differentiation of the skeletal cell populations. Stiffness of the scaffold can influence the mechanical environment in which cells are cultured, and it is predicted that increasing the stiffness of the scaffold enhances cartilage formation and reduces the amount of fibrous tissue formation in the osteochondral defect.^37^ Since stiffness of the Alvetex® 3-D scaffold has not been assessed in the current study, it is not possible to comment on the effect of scaffold stiffness and the influence of the mechanical environment on chondrogenic differentiation of the skeletal cells.

It is important to note that although expression of chondrogenic markers is significantly improved as a result of culture of STRO-1^+^ SSCs in the scaffolds, hypertrophic differentiation is not prevented. The results therefore suggest that bone marrow-derived STRO-1^+^ SSCs may indeed differ from articular chondrocytes with respect to their ultimate cellular fate, i.e. STRO-1^+^ SSCs adopt a transient rather than stable articular chondrocyte phenotype and undergo terminal differentiation. In order to maximise the potential of this adult stem cell population for cartilage repair, culture of SSCs in 3-D scaffolds with interconnected highly porous architecture should be complemented by improvements/modifications to existing protocols of *in vitro* chondrogenesis, specifically to prevent the phenotypic drift towards hypertrophic differentiation. This can include strategies such as locking SSCs in the desired differentiation state, for example, by establishment of DNA methylation patterns to silence the expression of hypertrophic genes, or co-culture of HACs and SSCs in 3-D chondroinductive low oxygen tension environments to reduce the incidence of hypertrophy by promoting cross-talk between the two skeletal cell populations, or application of mechanical loading to improve cartilage formation and limit hypertrophy.

In addition to the multipotent SSC subpopulation, the STRO-1^+^ population of human bone marrow contains osteoprogenitor cells, a significant proportion of glycophorin A-expressing nucleated erythroid cells and a small subset of B-lymphocytes.^24,30,38^ It is therefore not possible to obtain a homogenous population comprising solely of SSCs by using the STRO-1 antibody alone. A cell sorting approach based on positive selection for the antigen recognised by STRO-1 and negative selection for glycophorin A would be an effective initial strategy to minimise the heterogeneity of the STRO-1^+^ cell population and optimise the cellular starting material for tissue engineering. Additionally, a number of studies have shown that CD271^+^, CD146^+^, CD105^+^ and CD29^+^ mesenchymal stem cells (MSCs) exhibit higher chondrogenic potential in comparison to other MSC subpopulations, suggesting that these cell-surface antigens can be effectively used to isolate MSC subpopulations characterised by robust chondrogenic potential.^39–42^ Thus, an improved SSC enrichment strategy in the form of positive selection for the antigen recognised by STRO-1 and CD271/ CD146/ CD105/CD29, in combination with negative selection for glycophorin-A, would facilitate isolation of relatively homogenous subpopulations of SSCs (e.g. STRO-1^+^CD271^+^glycophorin A^−^ SSCs) from the heterogeneous STRO-1^+^ population that have enhanced chondrogenic potential and are suitable for bioengineering cartilage.

## Conclusion

The present study has demonstrated that monolayer-expanded HACs possess excellent chondrogenic potential in both “scaffold-free” pellet culture and culture using 3-D scaffolds. In contrast, STRO-1^+^ SSCs exhibit significantly lower chondrogenic differentiation potential in “scaffold-free” pellet culture. Although the application of highly porous 3-D scaffolds with interconnected pore network can improve the expression of chondrogenic markers by STRO-1^+^ SSCs, this is unable to prevent the phenotypic drift of the SSC population towards hypertrophy. Development of appropriate strategies to prevent hypertrophic differentiation is therefore a crucial step in delivering improved chondrogenic cell therapies from adult SSCs.
